# HyperART: non-invasive quantification of leaf traits using hyperspectral absorption-reflectance-transmittance imaging

**DOI:** 10.1186/s13007-015-0043-0

**Published:** 2015-01-16

**Authors:** Sergej Bergsträsser, Dimitrios Fanourakis, Simone Schmittgen, Maria Pilar Cendrero-Mateo, Marcus Jansen, Hanno Scharr, Uwe Rascher

**Affiliations:** Institute for Bio- and Geosciences, IBG-2: Plant Sciences, Forschungszentrum Jülich GmbH, 52425 Jülich, Germany; Present address: Department of Crop Science, Technological Educational Institute of Crete, GR 71004 Heraklio, Greece; Present address: Institute of Viticulture, Floriculture and Vegetable Crops, Hellenic Agricultural Organization ‘Demeter’ (NAGREF), P.O. Box 2228, GR 71003 Heraklio, Greece; Present address: LemnaTec GmbH, Pascalstraße 59, 52076 Aachen, Germany

**Keywords:** Absorption, *Cercospora beticola*, Chlorophyll content, FieldSpec, FluoWat, Hyperspectral imaging, Non-invasive phenotyping, Reflectance, Transmittance, Imaging spectroscopy

## Abstract

**Background:**

Combined assessment of leaf reflectance and transmittance is currently limited to spot (point) measurements. This study introduces a tailor-made hyperspectral absorption-reflectance-transmittance imaging (HyperART) system, yielding a non-invasive determination of both reflectance and transmittance of the whole leaf. We addressed its applicability for analysing plant traits, i.e. assessing *Cercospora beticola* disease severity or leaf chlorophyll content. To test the accuracy of the obtained data, these were compared with reflectance and transmittance measurements of selected leaves acquired by the point spectroradiometer ASD FieldSpec, equipped with the FluoWat device.

**Results:**

The working principle of the HyperART system relies on the upward redirection of transmitted and reflected light (range of 400 to 2500 nm) of a plant sample towards two line scanners. By using both the reflectance and transmittance image, an image of leaf absorption can be calculated. The comparison with the dynamically high-resolution ASD FieldSpec data showed good correlation, underlying the accuracy of the HyperART system. Our experiments showed that variation in both leaf chlorophyll content of four different crop species, due to different fertilization regimes during growth, and fungal symptoms on sugar beet leaves could be accurately estimated and monitored. The use of leaf reflectance and transmittance, as well as their sum (by which the non-absorbed radiation is calculated) obtained by the HyperART system gave considerably improved results in classification of *Cercospora* leaf spot disease and determination of chlorophyll content.

**Conclusions:**

The HyperART system offers the possibility for non-invasive and accurate mapping of leaf transmittance and absorption, significantly expanding the applicability of reflectance, based on mapping spectroscopy, in plant sciences. Therefore, the HyperART system may be readily employed for non-invasive determination of the spatio-temporal dynamics of various plant properties.

**Electronic supplementary material:**

The online version of this article (doi:10.1186/s13007-015-0043-0) contains supplementary material, which is available to authorized users.

## Background

Breeding of stress resistant crops for sustainable agricultural purposes is coupled with the identification of targeted traits and their genetic background [1-4]. In this content, the development and application of non-invasive systems becomes of outmost importance for plant phenotyping applications [4,5]. For this purpose, imaging techniques hold a prominent position in estimating various plant characteristics, ranging from pigment content to infection from diseases.

Light arriving on the leaf surface can be reflected, transmitted, or absorbed by the leaf. Quantifying the spectral characteristics of these components, by employing hyperspectral cameras can be very informative for the physiological status of the plants under research. Such spectroscopic retrieval of plant traits is recently gaining attention in the phenotyping community [6,7], not only due to their explicit connection with the leaf function and composition, but also due to the prospect of applying the method as imaging spectroscopy. This methodology offers the acquisition of information for the plants under study, without making physical contact with them. For instance, by using reflectance data, chlorophyll content of *Arabidopsis thaliana* [8] and disease symptoms’ incidence of *Beta vulgaris* [9-13] were recently monitored. Previous studies, however, are mostly limited to find optimal reflectance wavelengths (combination of those in form of indices) or methods that use reflectance signatures related to leaf characteristics such as chlorophyll content, nutritional status, or infection by diseases. This may be explained by the limited possibilities to measure transmittance in a rapid fashion. Despite diverse attempts in this field the technical possibilities still stay unexhausted. Measurements based only on reflectance or only on transmittance obviously neglect a part of the existing spectral information. Currently there are no known imaging approaches that can measure reflectance and transmittance simultaneously and derive absorption of plant leaves. We hypothesize that plant properties will be better estimated when both reflectance and transmittance are taken into account. Their simultaneous quantification will be highly advantageous for academic and industry-oriented research purposes as demonstrated in our results presented below.

Currently available instruments to quantify hyperspectral plant reflectance and transmittance (e.g., integrating sphere and leaf clip) operate in the spot scale (i.e., a few cm^2^) [14-16]. Therefore, several spots per leaf need to be assessed, in order to have an average value that is representative for the whole leaf. In this way, scaling plant reflectance and transmittance measurements to the entire leaf level will enable a more comprehensive assessment of a larger number of plants. More importantly, whole-leaf measurements offer spatial resolution allowing non-invasive observation of changes in leaf tissue owing to stressors.

Here we present a novel tailor-made instrument, named HyperART (Hyper = hyperspectral and ART Absorption, Reflectance, Transmittance), which quantifies simultaneously reflectance and transmittance at the whole-leaf scale. Firstly, we compare the data, obtained by the newly-introduced equipment, with a well-established high-performance spot-scale device (ASD- FieldSpec equipped with FluoWat; [17,18]). The FluoWat leaf-clip provides the possibility to record both reflection and transmission spectra of a single-point (i.e., limited in size) on a leaf. Secondly, we evaluated whether *Cercospora beticola* disease progression, a wide-spread pathogen, is more accurately derived by using the *Cercospora* Leaf Spot Index (CLSI) calculated from both reflectance and transmittance, as compared to the estimation that is solely based on reflectance. The CLSI index is computed by using wavelengths that were identified to be sensitive to the *C. beticola* disease symptoms [13]. Further, we tested the additional advantage of the HyperART in the estimation of leaf chlorophyll content of four species (i.e., maize, barley, rapeseed, tomato), based on twenty two commonly-used optical indices.

This paper introduces a novel tailor-made device, called HyperART. It can measure simultaneously spatially- and spectrally- resolved reflection and transmission, allowing the calculation of the spectral absorption image. This additional layer of information is expected to improve the quantification of plant traits. The above-mentioned measurements were performed with a comparable accuracy as with clip-on devices. The HyperART system also provides spatial information that can be potentially used to quantify spatial or temporal dynamics of leaf tissue changes. This is important as almost all processes or changes occurring in a leaf do not take place simultaneously throughout the whole tissue, but have initial points, from where they start spreading. The two employed case studies highlight the possibilities and advantages of the instrument. These are mainly the improved accuracy of results and the upscaling from the spot to the leaf level, encouraging its employment in future phenotyping studies.

### HyperART a novel measurement system

The HyperART system is a novel imaging system developed for non-invasive evaluation of leaf properties, based on recordings of reflectance, transmittance and absorption. In contrast to commercially-available instruments that allow point measurements (i.e., limited to only few cm^2^), this new equipment offers mapping of the whole leaf. For highest precisions, a geometrically precise system and flat samples deserve careful consideration, as described below.

**Figure 1:**
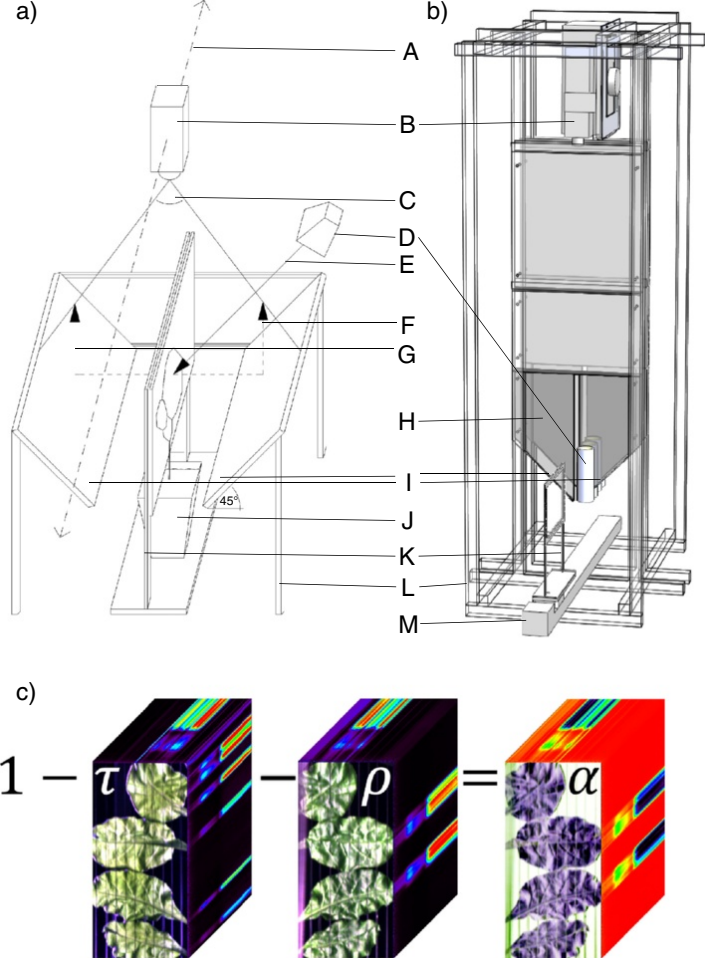
**Two prototypes of the HyperART system with an image example. (a, b)** Schematics of the hyperspectral absorption reflectance transmittance imaging (HyperART) system, employed for simultaneous recording of both reflectance (ρ) and transmittance (τ). In the first prototype **(a)** the camera is moving, whereas in the second one **(b)** the samples are moved. A, scan direction; B, line scanner; C, field of view; D, illumination source; E, light beam; F, reflected light; G, transmitted light; H, black painted metal sheets (to avoid direct illumination of the mirror from the light source and to reduce light scattering in the scanning process); I, mirrors; J, plant, of which leaves are fixed in the clip; K, clip, where leaf sample is placed; L, framework; M, slide bar. **(c)** Calculation of absorption (α) in the hyperspectral cube (acquired by the HyperART system), based on ρ and τ.

Currently, two prototypes of the HyperART system are available (Figure 1a, b). The first prototype (Figure 1a) was used for the first case study (sugar beet), while the second prototype (Figure 1b) was employed for the measurements of the second case study (rapeseed, tomato, maize and barley). The main components of both prototypes include: i) a custom-made rectangular clip to hold and flatten the leaves (Figure 1K), ii) two mirrors which redirect both the reflected and transmitted light (Figure 1I), iii) an illumination system (Figure 1D), iv) an imaging system, composed by two hyperspectral cameras (together offering a spectral range of 400–2500 nm; Figure 1B), and v) a slide bar which moves the imaging system (first prototype; Figure 1a A) or the custom-made rectangular clip holding the sample (second prototype; Figure 1b M). The main difference between the two prototypes relies on the position of the light source (relative to the mirror) and the scanning procedure (i.e., movement of the imaging system or the clip). The different components of the HyperART system will be described firstly, followed by its working principle.

#### Custom-made rectangular clip

It consisted of two frames that were connected at the top (Figure 1K). The frames had dimensions of length × width × depth equal to 32 × 41 × 0.5 cm (inner dimensions 28 × 20 cm), and can host samples as large as 560 cm^2^. The clip keeps the leaf samples as flat as possible. In the first prototype, the frames were made from metal. Moreover, a metal wire, at a distance of 1.5 cm in the horizontal direction of the frame, was used to position the leaves. The frames and the wire were painted with black mat colour (Dupli Color, MOTIP DUPLI GmbH, Haßmersheim, Germany). In the second prototype, the frames were manufactured from cast polymethylmethacrylat sheet (Plexiglas®GS, Evonik Industries AG, Essen, Germany), and were also painted with black mat colour (described above). Leaf positioning was performed by tight black fishing lines at a distance of 2 cm in both horizontal and vertical (frame) directions.

#### Mirror

A flat surface mirror (gertenbach.info e. K., Wölltein, Germany) was employed, having a minimum of 85% spectral reflectance performance (in the range of 400–700 nm) at 45° angle relative to illumination. The first prototype contains two (rectangular) mirrors with dimensions of length × width × depth equal to 28 × 20 × 0.5 cm. The second prototype employs four (rectangular) mirrors with the following dimensions: length × width × depth equal to 25.5 × 0.5 × 0.3 cm. In both prototypes, the mirrors were placed exactly at 45° relative to both the leaf clip (thus the leaf sample) and camera planes.

#### Illumination system

In the first prototype, sample illumination was offered by two double-ended halogen lamps (OMNILUX® 230 V/4000 W R7s 118 mm, OMNILUX, Waldbüttelbrunn, Germany), placed in a halogen spotlight reflector. In the second prototype, similar lamps (OMNILUX® 230 V/120 W R7s 117 mm, OMNILUX, Waldbüttelbrunn, Germany) were placed into a custom made tube reflector from a polished-sheet metal. The lamps were covered with frosted quartz glass, which diffuses the light in order to avoid shadows and enhance the quality of the acquired hyperspectral data. A custom-made converter was employed to transform (rectify) alternating current to direct current (residual ripple ≤ 5%). This was a three-phase power supply unit, manufactured by a company (J.Schneider Elektrotechnik GmbH, Offenburg, Germany). Direct current was preferable for obtaining hyperspectral data by using a line scanner, since it prevents the fluctuation of the halogen lamp emission, which would take place under alternating current. In both prototypes, the lamps were placed in order to illuminate one side of the leaves.

#### Hyperspectral imaging system

The HyperART system hosted two hyperspectral cameras employed on sequence, and together offering a spectral range between 400 and 2500 nm. Both devices work as push broom line scan cameras. The first hyperspectral camera (PS V10E, Spectral Imaging Ltd., Oulu, Finland) employs a high speed interlaced CCD detector. It is sensitive in the 400 to 1000 nm range, and offers a spatio-spectral resolution of 1392 × 1040 pixels. The nominal spectral resolution is 2.8 nm full width half maximum (FWHM). The spectral sampling interval depends on the spectral binning and varies between 0.63 and 5.06 nm. In our experiments with the first prototype, a binning of 4 (spectral resolution) and 1 (spatial resolution) was employed. In the experiments with the second prototype, a binning of two (spectral resolution) by two (spatial resolution) was employed. The second hyperspectral camera (SWIR, Spectral Imaging Ltd., Oulu, Finland) uses a cooled, temperature-stabilized MCT detector, which is sensitive to the spectral range between 1000 and 2500 nm. The sensor can acquire 320 spatial pixels’ and 256 spectral bands’ images. The spectral sampling rate is 6.3 nm, combined with a nominal spectral resolution of 10 nm (FWHM).

#### Scanning procedure

At the first prototype, the two hyperspectral cameras were fixed on a slide bar (BiSlide, Velmex Inc., Bloomfield, NY, USA) that moves along an 1.5 m path at a constant speed (≈15 mm sec^−1^). The cameras were moved in parallel to the middle line and between the opposite standing mirrors. As the hyperspectral cameras were moved along the slide bar, they recorded the reflected and transmitted light of the whole leaf sample, which was placed in the clip, as explained above. In the second prototype, the hyperspectral cameras were fixed on a framework over the middle point and between the opposite standing mirrors (Figure 1b L). In this case, the clip (holding the leaf sample) was moved along the middle line, between the mirrors that were fixed on the slide bar (Figure 1b M).

The working principle of the HyperART system was identical for both prototypes, and enabled the upward redirection of transmitted and reflected light from the leaf sample towards the hyperspectral camera system through the mirrors. Thus, the unique advantage of the HyperART system is that a reflectance and transmittance image of a leaf can be recorded simultaneously by a single hyperspectral camera. For measurements, leaves must be positioned into the clip (Figure 1K), which can be done without excising them from the plant, since the pot (≤17 cm height) can be placed below the clip frame (Figure 1a J). Placing the leaves between the frames directs the leaf surface into a flat plane. Inside the clip, the leaves were illuminated from one side, while the leaf was positioned between the two mirrors (Figure 1I). Each mirror faced a different side of the clip (thus the sample). In the first prototype, the light source was placed behind one mirror, and opposite to the sample at an angle of 45° (Figure 1a D). In the second prototype, the light source was placed from the sides of mirrors (Figure 1b D) that allowed nearly direct illumination of the sample. The mirrors posed both the clip window with the sample, and the camera, at a 45° angle. One mirror redirected the reflected light (Figure 1a F) to the two line scanners (i.e., upwards; Figure 1B), while the other mirror did the same for the transmitted light (Figure 1a G). The distance of the upper mirror edge to the two line scanners was set to 0.85 m for both prototypes.

To determine the incoming electromagnetic radiation by the first prototype, a 99% reflective white standard (Spectralon, Labsphere Inc., North Dutton, NH, USA) was placed instead of the clip. This was done before and following the measurements. In the second prototype, the transmitted light was determined by placing a white diffuser lambertian transmission sheet (Zenith Polymer® ≈ 50% transmission, SphereOptics GmbH, Uhldingen, Germany) between the mirrors.

### Image processing

Hyperspectral images were processed by using ENVI 4.8 (Exelis Visual Information Solutions, Inc., Boulder, CO, USA) and MatLab R2012b (MathWorks®, Natick, Massachusetts, USA) Software. Different protocols were used for image pre-processing obtained from the first and second prototype.

#### First prototype

Firstly, a dark frame image was subtracted from both the leaf and white reference images (Fastspec extension of ENVI). Secondly, images were cut to separate the reflection and transmission images. The transmission image was flipped to overlay the reflection image, and co-registered by the corresponding points in order to correct slight distortions, produced by image acquisition (VLFeat toolbox in MatLab; [19]). In the next step, each image pixel was divided by the corresponding pixel within the white reference image, and multiplied by a correction factor for white reference material (provided by Labsphere Inc.), aiming at both correcting for illumination differences and achieving relative values (Spectral Math tool of ENVI).

#### Second prototype

In this prototype images needed modified processing steps since the scan procedure was different as compared to the first prototype. Additionally to the white standard, we here measured a white diffuser lambertian transmission sheet, before scanning the plant samples. By mosaicking both white references we got a white reference image with the same numbers of lines as the sample image. The sample image was normalized by this white reference image for reflection and transmission sides line by line using a normalisation tool from SPECIM (Spectral Imaging Ltd., Oulu, Finland) implemented in ENVI. The transmission image was then flipped and co-registered. With the objective to obtain correct relative values, a correction factor was applied to the reflection and transmission images (provided by Labsphere Inc. and SphereOptics GmbH, respectively).

#### Absorption mapping

**Figure 2:**
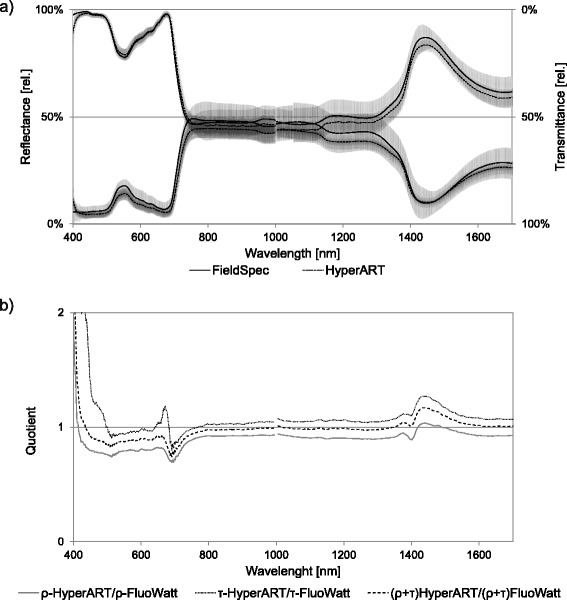
**Comparison of spectral signatures obtained by FluoWat and HyperART devices. (a)** Reflectance (ρ) and transmittance (τ) spectra of sugar beet leaves acquired by using FluoWat or the hyperspectral absorption reflectance transmittance imaging (HyperART) system. Data are expressed as relative values. Dashed areas indicate SEM (*n* = 5). **(b)** Quotients of ρ, τ and sum of ρ with τ (i.e., ρ + τ) of spectral data acquired by using FluoWat and the HyperART system.

Based on the assumption that the sum of absorption (α), reflectance (ρ) and (flipped) transmittance (τ) equals to 1 (Figure 2), α was calculated by subtracting ρ and τ from the unit (i.e., α = 1 – ρ + τ) for images acquired by using both prototypes. The α contains the information about leaf components and is not sensitive to light scattering effects, caused by leaf surface properties [20]. However, it is not practical to apply different vegetation indices on α, because these indices were developed for approaches based on ρ measurements. To highlight the advantage of the newly-introduced HyperART system and make the data comparison clear, a ρ comparable spectral signature ought to be employed. Therefore, the non-absorbed radiation ρ + τ images (that are equal to inverted α because ρ + τ = 1 – α) were used in analysis.

## Results and discussion

### Validation of HyperART spectral data

Reflectance and transmittance spectral signatures of sugar beet leaves were recorded by using either the HyperART system or the FieldSpec spectoradiometer, equipped with a FluoWat device (Figure 2a). In order to validate the HyperART-obtained measurements, reflectance (ρ), transmittance (τ), and the sum of reflectance and transmittance spectra (non-absorbed radiation, ρ + τ) were compared to the FluoWat spectra (Figure 2).

A similar ρ and τ spectrum acquired by either device was found (Figure 2a). Over the full ρ spectral range (except 1400–1500 nm), relative values acquired by the HyperART system were 1–5% lower than those of FluoWat. For the τ spectra, the HyperART system data yielded either higher (>800 nm) or lower (550 nm) values than those of the FluoWat data. However, the difference in the τ spectra was minor (≈1%), as compared to the difference in the ρ spectrum. In the FluoWat device, there was an overlap between the ρ and τ spectra at the near infrared region (NIR, 736–1100 nm) (Figure 2a). HyperART ρ and τ spectra were overlapping in the 1012–1031 nm region. Because of this overlap, the normalized α spectrum is not presented. The noted differences between the values, obtained by the two devices, might be due to the fact that the leaf regions measured by the FluoWat device, are not exactly identical as the selected (for comparison) areas in the images obtained by using the HyperART system. The gap of the values observed at 1000 nm at both ρ and τ spectra, is due to the configuration of the FieldSpec and the hyperspectral imaging system. Both devices combine two different sensors, where the first one covers the region up to 1000 nm, while the second one includes longer wavelengths (i.e., ≥ 1000 nm).

For the ρ, τ, and ρ + τ HyperArt normalized spectra (Figure 2b) the highest deviations were observed in the photosynthetic active radiation region (PAR, 420–730 nm) and around the water absorption band at 1450 nm. For the whole spectrum (420–1700 nm), standard deviations of 0.07 (ρ), 0.19 (τ) and 0.07 (ρ + τ) were found. These low standard deviation values indicate that the data obtained with the HyperART system are comparable to those taken by using FluoWat.

The coupling of two hyperspectral cameras (i.e., VNIR and SWIR) might be important for future work, where additional traits, from those studied here, are of interest. For instance, the spectral region, offered by the SWIR camera (i.e., 1000–2500 nm), is often employed to detect senescence and water stress symptoms [21,22]. We were able to show that the HyperART system performs well with the SWIR camera, though its potential in detecting plants characteristics is not dealt in the current study. The SWIR images and the VNIR images can be matched together using corresponding points. This matching of VNIR and SWIR images may assist extended spectral signature for every pixel in only one image. However the spatial resolution in the VNIR image needs to be adjusted to the SWIR image or vice versa.

### *Cercospora beticola* symptoms

**Figure 3:**
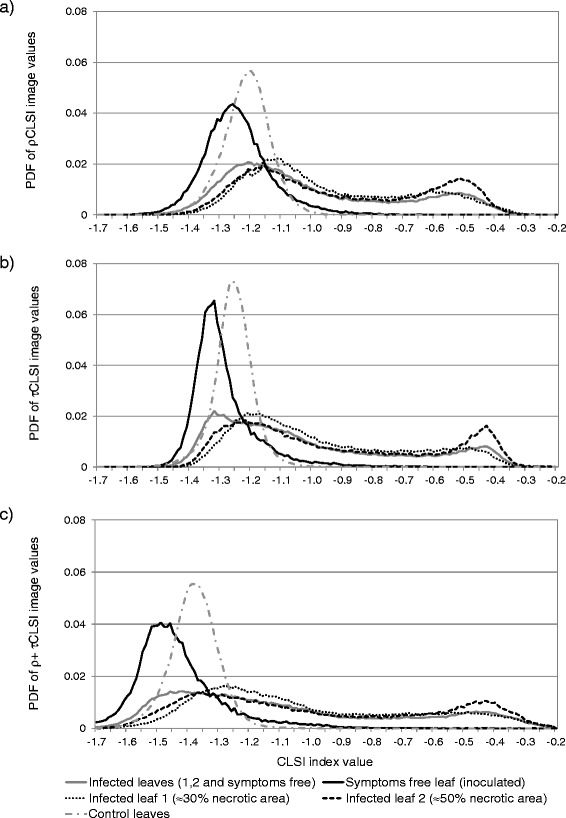
**Values difference in normalized histograms of Cercospora Leaf Spot Index (CLSI) images.** Normalized histograms (estimated probability functions PDF) of the calculated CLSI values based on **(a)** reflectance (ρ), **(b)** transmittance (τ) or **(c)** combination of ρ with τ (i.e., ρ + τ). Spectra were acquired by using the hyperspectral absorption, reflectance, transmittance (HyperART) imaging system. Measurements were conducted on three leaves of an infected sugar beet plant, and on three leaves of another non-infected (control) sugar beet plant. The two leaves of the former plant showed visual symptoms of infection, whereas the third one was symptom-free.

**Figure 4:**
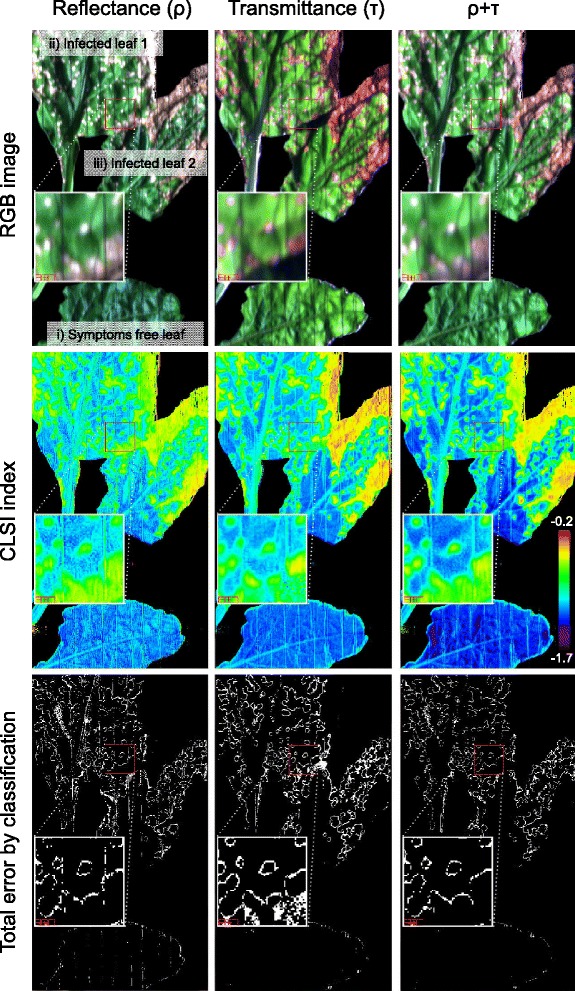
**RGB, colour coded** ***Cercospora*** **Leaf Spot Index (CLSI) and total classification error images.** Images were calculated from reflectance (ρ), transmittance (τ) and combination of ρ with τ (i.e., ρ + τ) of sugar beet leaves infected by *Cercospora beticola*. The classification was performed by supervised SVM (support vector machine) classification on hyperspectral images, and unsupervised K-Mean (on CLSI images) using two classes (visible symptoms and plat tissue without visible symptoms). Images were acquired by using the hyperspectral absorption reflectance transmittance imaging (HyperART) system. The rectangular panels in the images show the enlargement of red bordered infected leave surface.

Following the data comparison between the HyperART system and FluoWat device we tested if the HyperART is suitable for the detection of *Cercospora* leaf spot disease. Therefore normalized histograms (Figure 3) of the CLSI images with the leaves of one infected and one non-infected sugar beet plant were analysed and compared among each other. Additionally the precision of the unsupervised classification on the ρ, τ and ρ + τ CLSI images of the infected leaves were verified (Figure 4).

Three leaves of the infected sugar beet plant (shown in Figure 4) can be described by visual inspection as: i) symptom-free leaf (lower leaf in Figure 4), ii) leaf with ≈ 30% infected area (upper leaf in Figure 4), and iii) leaf with ≈ 50% necrotic area (middle leaf in Figure 4). In order to assess the CLSI value changes induced by pathogen infection, the CLSI histogram from three leaves of the non-infected control plant was also computed.

The CLSI shows similar bimodal distribution when calculated from ρ (ρCLSI), τ (τCLSI), or ρ + τ (ρ + τCLSI) images of leaves with infection symptoms. The normalized histogram of the leaves attached on the control plant, or the symptom-free leaf, attached on the infected plant, were clearly different (Figure 3). Furthermore there is a difference between the normalized histogram of inoculated but still symptom-free leaf and the normalized histogram of control leaves. They both show a single maximum only, however, the distribution of the symptom-free leaves show a heavy right tail, whereas the control leaves are normally distributed. As a right tail is an index shift towards infection symptoms, this indicates that pre-symptomatic fungal development changed leaf spectral properties. Therefore the CLSI offers a perspective for using the method in early detection of fungal infection stages.

The normalized histograms of the symptomatic leaves showed a shift from the right peak that is related to the degree of leaf infestation. While the normalized ρ and ρ + τ histograms are Gaussian-like, the normalized τ histograms were sharper, indicating a lower noise influence. Peak locations varied for the different measurement modalities. The distance between the peak locations were larger in τCLSI and largest in ρ + τCLSI compared to ρCLSI. This might enable a better assignment of the pixels into the categories infected or non-infected. The bimodal amplitude (A_b_) was equal to 0.45, 0.48 and 0.43 for ρCLSI, τCLSI and ρ + τCLSI, respectively, being in accordance with our observation of sharper peaks in the normalized τCLSI-histogram. Considering all CLSI histograms, it can be stated that over 90% of the pixels in the index images were distributed between −1.7 and −0.2. Higher values indicate increasing disease severity and vice-versa.

The computed CLSI value range in this study differs due to variation in plant properties and measurement method from the range (−0.45 to 0.3) reported in the literature [13,23]. For instance, leaf pigment content, as well as leaf internal and external structure have been shown to affect plant reflectance properties [24]. In addition, illumination conditions (e.g., shadow or illumination angle) during measurement or measurement methodology (e.g., point measurements, leaf scale or canopy scale) can also raise differences in the CLSI values’ range. The differences in CLSI value may be explained by the use of 698 nm and 734 nm wavelengths in CLSI equation. These wavelengths are located in the red edge spectral region, and are sensitive to chlorophyll content [25-28]. The sensitivity at 698 nm is translated to decreased reflectivity (thus lower CLSI values in the normalized part of equation), as chlorophyll content increases. When we subtract lower reflectance value (due to chlorophyll absorption at 734 nm) from the normalised part of equation then this might produce slightly higher CLSI values of the leaves attached on the control plant, as compared to the symptom-free leaf attached to the infected plant.

To investigate the classification performance, the tissue having CLSI values lower than the lower peak center was categorized as ‘non-infected’ (≈27%, 14%, and 21% of the pixels for ρCLSI, τCLSI, and ρ + τCLSI, respectively), while tissue giving higher values than the higher peak center is termed as ‘infected’ (≈14%, 19%, and 36% of the pixels for ρCLSI, τCLSI, and ρ + τCLSI, respectively). The remaining pixels (≈59%, 67%, and 43% for ρCLSI, τCLSI, and ρ + τCLSI, respectively) between the peaks allow the presence of classification errors. This observation leads to the conclusion that the separation of infected from non-infected leaf tissue is better done with the ρ + τCLSI image, since most of the tissue area is unambiguously classified.

In the RGB τ image, the reddish colour may be an indication of anthocyanin production in the (sugar beet) leaves, due to infection (Figure 4). The CLSI images have a rainbow colour code, ranging from −1.7 (blue) to −0.2 (red) (Figure 4), which is similar to the histograms in Figure 3. The ρCLSI image seems to have a slightly higher noise level, as compared to the other images. The τCLSI image shows a better contrast between extremes, while the ρ + τCLSI image differentiates the initial symptom and its growing borders. The unsupervised classification yields 93.4%, 92.3% and 94.7% overall accuracy (automatically calculated in ENVI using contingency matrix which did compare supervised classification with unsupervised classification) for the whole ρCLSI, τCLSI and ρ + τCLSI images respectively, being well in accordance with our previous observation. In total, the ρCLSI index classification quantifies 33.9% of the tissue as infected (i.e., 66.1% non-infected), while the τCLSI index classification led to an estimation of 29.3% infected tissue area (i.e., 70.7% non-infected). The ρ + τCLSI image classification results in 31.8% infected leaf area, and gave the best overall accuracy, as explained below.

The total classification error images (Figure 4) suggest that the noise in the ρCLSI image leads to an overestimation of infected area. This overestimation may be due to the leaf veins or shadow parts, as a result of leaf waviness. Another error source is an overlay of leaves by the black wire taut in the leaf clip. The opposite is noted for the τCLSI image classification, where the non-infected area seems to be overestimated. This overestimation of the non-infected area might be due to both the overlapping region between the two leaves, which were together placed in the clip, and an underestimation of the symptom border. The τCLSI was found to feature comparably low noise, being also in accordance with the sharper peaks in the respective histogram (Figure 3). The ρ + τCLSI image gave the best class separation, supressed noise and counted overlapping regions that led to the most precise estimation of disease severity. In the ρ + τCLSI image, the unsupervised classification gave a 99.4% of non-infected tissue area in the symptom-free leaf. The infected leaves were classified to have 38.7% and 44.6% infected areas, respectively.

In general, our visual estimation had an error between 5.4% and 8.7%, as compared to the image classification results. Therefore, the machine-based classification method (in our case the HyperART system) was found to be more precise and reliable in quantifying disease severity, as compared to assessments based on visual inspection [29].

### Leaf chlorophyll content estimation

Leaves of four species (maize, rapeseed, barley and tomato), grown under two different nitrogen regimes, were analysed for chlorophyll content. The relationships between 22 optical indices (summarized in Table 1), calculated from 3 source HyperART images (ρ, τ, and ρ + τ) and destructively-assessed chlorophyll content were analysed (i.e., 66 indices = 22 optical indices × 3 HyperART source images). Both logarithmic (Table 2) and linear (Table 3) regression models were employed for the analysis.

**Table 1:** **Formulas and abbreviations of the employed vegetation indices**

**Index**	**Equation**	**Indicator (Scale)**	**Reference**
Area under continuum-removed curve	ANCB_650 − 720_ : Continuum removal based method	Chlorophyll a & b content (Canopy)	[30]
Blue/Green index	${\mathrm{BGI}}_2=\frac{\mathrm{R}450}{\mathrm{R}550}$	Chlorophyll content (Canopy)	[31]
Chlorophyll green index	$\mathrm{Chlgreen} = \frac{\mathrm{R}790}{\mathrm{R}550} - 1$	Chlorophyll (Leaf)	[32]
Chlorophyll red edge index	$\mathrm{Chlred}\ \mathrm{edge} = \frac{\mathrm{R}790}{\mathrm{R}705} - 1$	Chlorophyll (Leaf)	[32]
Enhanced vegetation index	$\mathrm{E}\mathrm{V}\mathrm{I}=2.5\kern0.5em *\frac{\mathrm{R}800-\mathrm{R}670}{\mathrm{R}800+6\kern0.5em *\kern0.5em \mathrm{R}670-7.5\kern0.5em *\kern0.5em \mathrm{R}400+1}$	Chlorophyll (Canopy)	[33]
Modified chlorophyll absorption reflectance index	$\mathrm{MCARI} = \left(\left(\mathrm{R}701 - \mathrm{R}670\right) - 0.2\kern0.5em *\kern0.5em \left(\mathrm{R}701 - \mathrm{R}550\right)\right)\kern0.5em *\kern0.5em \frac{\mathrm{R}701}{\mathrm{R}670}$	Chlorophyll (Leaf, Canopy)	[34]
Modified normalized difference index	$\mathrm{m}\mathrm{N}\mathrm{D} = \frac{\mathrm{R}750-\mathrm{R}705}{\mathrm{R}750+\mathrm{R}705-2\kern0.5em *\kern0.5em \mathrm{R}445}$	Chlorophyll (Leaf)	[35]
Modified simple ratio	$\mathrm{m}\mathrm{S}\mathrm{R} = \frac{\mathrm{R}750 - \mathrm{R}445}{\mathrm{R}705 - \mathrm{R}445}$	Green biomass Chlorophyll (Leaf)	[35]
Normalized difference index	$\mathrm{N}\mathrm{D} = \frac{\mathrm{R}750 - \mathrm{R}705}{\mathrm{R}750 + \mathrm{R}705}$	Chlorophyll (Leaf)	[36]
Normalized difference vegetation index	$\mathrm{NDVI}=\frac{\mathrm{R}800-\mathrm{R}670}{\mathrm{R}800+\mathrm{R}670}$	Biomass, leaf area (Canopy)	[37]
Structure insensitive pigment index	$\mathrm{SIPI}=\frac{\mathrm{R}800-\mathrm{R}455}{\mathrm{R}800+\mathrm{R}680}$	Carotinoid/chlorophyll a ratio (Leaf)	[38]
Simple ratio 1	${\mathrm{SR}}_{750/710} = \frac{\mathrm{R}750}{\mathrm{R}710}$	Chlorophyll (Canopy)	[39]
Simple ratio 2	$\mathrm{S}\mathrm{R} = \frac{\mathrm{R}800}{\mathrm{R}670}$	Green biomass (Canopy)	[40]
Pigment specific normalized difference a	$\mathrm{PSNDa}=\frac{\mathrm{R}800-\mathrm{R}680}{\mathrm{R}800+\mathrm{R}680}$	Chlorophyll a (Leaf)	[41]
Pigment specific normalized difference b	$\mathrm{PSNDb} = \frac{\mathrm{R}800 - \mathrm{R}635}{\mathrm{R}800 + \mathrm{R}635}$	Chlorophyll b (Leaf)	[41]
Plant senescence index	$\mathrm{PSRI} = \frac{\mathrm{R}680 - \mathrm{R}500}{\mathrm{R}750}$	Plant senescence (Leaf)	[42]
Pigment specific simple ratio a	$\mathrm{PSSRa}=\frac{\mathrm{R}800}{\mathrm{R}680}$	Chlorophyll a (Leaf)	[41]
Pigment specific simple ratio b	$\mathrm{PSSRb}=\frac{\mathrm{R}800}{\mathrm{R}635}$	Chlorophyll b (Leaf)	[41]
Transformed chlorophyll absorption in reflectance Index	$\mathrm{TCARI}=3*\left(\left(\mathrm{R}700-\mathrm{R}670\right)-0.2*\left(\mathrm{R}700-\mathrm{R}550\right)*\left(\frac{\mathrm{R}700}{\mathrm{R}670}\right)\right)$	Chlorophyll (Canopy)	[43]
Transformed chlorophyll absorption in reflectance Index/Optimized soil-Adjusted vegetation index	$\frac{\mathrm{TCARI}}{\mathrm{OSAVI}}=3\kern0.5em *\kern0.5em \frac{\left(\mathrm{R}700-\mathrm{R}670\right)-0.2\kern0.5em *\kern0.5em \left(\mathrm{R}700-\mathrm{R}550\right)\kern0.5em *\kern0.5em \left(\frac{\mathrm{R}700}{\mathrm{R}670}\right)}{\left(1+0.16\right)\kern0.5em *\kern0.5em \frac{\mathrm{R}800-\mathrm{R}670}{\mathrm{R}800+\mathrm{R}670+0.16}}$	Chlorophyll (Canopy)	[43]
Triangular vegetation index	TVI = 0.5 * (120 * (R750 − R550) − 200 * (R670 − R550))	Leaf area and chlorophyll content (Canopy)	[44]
Vogelmann	$\mathrm{V}\mathrm{O}\mathrm{G}=\frac{\mathrm{R}740}{\mathrm{R}720}$	Chlorophyll (Leaf)	[45]

The scale (leaf or canopy), at which these are commonly used, is also presented.

**Table 2:** **Vegetation indices performance based on a logarithmic regression model**

**Index**	**Rank**	**Source**	**COR**	**R** ^**2**^	**F**	**P**	**Index**	**Rank**	**Source**	**COR**	**R** ^**2**^	**F**	**P**
ANCB	8	***ρ +*** ***τ***	0.80	0.64	66.94	0.00	PSNDb	7	***τ***	0.80	0.64	67.86	0.00
	13	***τ***	0.77	0.60	56.98	0.00		20	***ρ + τ***	0.75	0.56	48.48	0.00
	26	**ρ**	0.71	0.50	37.60	0.00		43	ρ	0.55	0.30	16.15	0.00
BGI2	9	**ρ**	0.80	0.63	65.65	0.00	PSRI	53	τ	0.37	0.14	6.02	0.02
	27	τ	−0.69	0.48	34.56	0.00		54	ρ + τ	0.35	0.12	5.41	0.03
	48	ρ + τ	0.46	0.22	10.41	0.00		61	ρ	0.15	0.02	0.82	0.37
Chlg	38	ρ + τ	0.60	0.36	21.10	0.00	PSSRa	23	***τ***	0.74	0.55	45.88	0.00
	41	ρ	0.56	0.31	17.32	0.00		49	ρ + τ	0.45	0.20	9.49	0.00
	57	τ	0.25	0.06	2.58	0.12		65	ρ	0.08	0.01	0.23	0.64
Chlre	15	***τ***	0.77	0.59	54.90	0.00	PSSRb	12	***τ***	0.78	0.61	58.42	0.00
	22	***ρ + τ***	0.75	0.56	47.42	0.00		21	***ρ + τ***	0.75	0.56	48.36	0.00
	33	ρ	0.63	0.40	25.60	0.00		51	ρ	0.41	0.17	7.64	0.01
EVI	1	***ρ + τ***	0.85	0.72	99.16	0.00	SR1	10	***τ***	0.80	0.63	65.28	0.00
	34	τ	0.62	0.38	23.21	0.00		17	***ρ + τ***	0.76	0.58	53.06	0.00
	44	ρ	0.54	0.29	15.88	0.00		31	ρ	0.66	0.44	30.07	0.00
MCARI	36	ρ	−0.61	0.37	22.07	0.00	SIPI	24	***τ***	0.72	0.52	41.39	0.00
	46	τ	0.51	0.26	13.02	0.00		52	ρ + τ	0.38	0.14	6.38	0.02
	47	ρ + τ	−0.49	0.24	12.24	0.00		66	ρ	−0.03	0.00	0.03	0.86
mND	3	***τ***	0.83	0.69	85.43	0.00	SR2	18	***τ***	0.76	0.58	51.62	0.00
	4	***ρ + τ***	0.82	0.67	76.13	0.00		45	ρ + τ	0.53	0.29	15.16	0.00
	28	ρ	0.69	0.47	34.36	0.00		63	ρ	0.10	0.01	0.42	0.52
mSR	14	τ	0.77	0.60	56.46	0.00	TCARI	37	ρ	−0.60	0.36	21.16	0.00
	40	ρ + τ	−0.57	0.32	18.27	0.00		42	ρ + τ	−0.56	0.31	17.09	0.00
	56	ρ	−0.29	0.08	3.46	0.07		64	τ	0.09	0.01	0.32	0.57
ND	2	***τ***	0.84	0.70	90.52	0.00	TCARI/OSAVI	30	ρ	−0.67	0.44	30.36	0.00
	6	***ρ + τ***	0.80	0.65	69.52	0.00		32	ρ + τ	−0.66	0.43	29.11	0.00
	29	ρ	0.69	0.47	34.36	0.00		59	τ	−0.21	0.04	1.71	0.20
NDVI	16	***τ***	0.77	0.59	54.01	0.00	TVI	50	ρ + τ	0.43	0.19	8.68	0.01
	35	ρ + τ	0.62	0.38	23.20	0.00		55	τ	0.29	0.09	3.57	0.07
	58	ρ	0.23	0.05	2.16	0.15		62	ρ	0.14	0.02	0.81	0.37
PSNDa	19	***τ***	0.76	0.57	50.71	0.00	VOG	5	***τ***	0.82	0.66	75.39	0.00
	39	ρ + τ	0.57	0.33	18.40	0.00		11	***ρ + τ***	0.78	0.61	60.69	0.00
	60	ρ	0.18	0.03	1.22	0.28		25	ρ	0.71	0.50	37.91	0.00

Determination (R^2^) and correlation (COR) coefficients, as well as significance level of the relation between the 22 vegetation indices (abbreviations in Table 1) and destructively-measured chlorophyll content. Vegetation indices were calculated based on reflectance (ρ), transmittance (τ) or combination of ρ with τ (i.e., ρ + +τ) source images. Indices were ranked based on the R^2^ value (all three source images were included in the ranking). The entire leaf surface was averaged (n = 40). All four species (maize, rapeseed, barley and tomato) were plotted together (examples are shown in Additional file 1: Figure S1). Plants were grown under control or deficient nitrogen levels. Bold text indicates R^2^ values greater or equal to 0.5.

**Table 3:** **Vegetation indices performance based on a linear regression model**

**Index**	**Rank**	**Source**	**COR**	**R** ^**2**^	**F**	**P**	**Index**	**Rank**	**Source**	**COR**	**R** ^**2**^	**F**	**P**
ANCB	12	***τ***	0.75	0.56	49.14	0.00	PSNDb	17	***τ***	0.73	0.53	42.17	0.00
	15	***ρ + τ***	0.74	0.55	45.75	0.00		23	ρ + τ	0.65	0.43	28.49	0.00
	28	ρ	0.62	0.39	24.20	0.00		49	ρ	0.45	0.20	9.53	0.00
BGI2	5	**ρ**	0.78	0.61	59.78	0.00	PSRI	52	τ	0.34	0.12	4.97	0.03
	31	τ	−0.60	0.36	21.79	0.00		56	ρ + τ	0.27	0.07	3.05	0.09
	42	ρ + τ	0.50	0.25	12.68	0.00		64	ρ	0.05	0.00	0.09	0.77
Chlg	37	ρ + τ	0.55	0.30	16.15	0.00	PSSRa	18	***τ***	0.71	0.51	39.52	0.00
	43	ρ	0.49	0.24	11.86	0.00		50	ρ + τ	0.37	0.14	6.16	0.02
	57	τ	0.23	0.05	2.12	0.15		66	ρ	0.02	0.00	0.02	0.88
Chlre	13	***τ***	0.75	0.56	47.68	0.00	PSSRb	9	***τ***	0.76	0.58	52.50	0.00
	19	***ρ + τ***	0.71	0.50	38.59	0.00		20	ρ + τ	0.70	0.48	35.76	0.00
	34	ρ	0.59	0.34	19.80	0.00		53	ρ	0.33	0.11	4.67	0.04
EVI	1	***ρ + τ***	0.83	0.70	87.37	0.00	SR1	6	***τ***	0.78	0.60	57.58	0.00
	24	τ	0.65	0.42	27.30	0.00		16	***ρ + τ***	0.73	0.54	43.81	0.00
	40	ρ	0.52	0.27	13.72	0.00		29	ρ	0.62	0.39	23.97	0.00
MCARI	35	ρ	−0.58	0.34	19.23	0.00	SIPI	30	τ	0.61	0.37	22.38	0.00
	39	ρ + τ	−0.52	0.27	14.32	0.00		55	ρ + τ	0.29	0.08	3.39	0.07
	48	τ	0.46	0.21	10.01	0.00		63	ρ	−0.08	0.01	0.27	0.60
mND	4	***τ***	0.80	0.64	67.83	0.00	SR2	14	***τ***	0.74	0.55	45.79	0.00
	7	***ρ + τ***	0.77	0.59	55.58	0.00		47	ρ + τ	0.46	0.21	10.05	0.00
	26	ρ	0.63	0.39	24.65	0.00		65	ρ	0.04	0.00	0.06	0.81
mSR	10	**τ**	0.75	0.57	49.85	0.00	TCARI	38	ρ	−0.54	0.29	15.61	0.00
	33	ρ + τ	−0.59	0.35	20.20	0.00		45	ρ + τ	−0.48	0.23	11.14	0.00
	54	ρ	−0.31	0.10	4.15	0.05		60	τ	0.13	0.02	0.67	0.42
ND	3	***τ***	0.80	0.64	68.15	0.00	TCARI/OSAVI	32	ρ	−0.59	0.35	20.27	0.00
	11	***ρ + τ***	0.75	0.56	49.16	0.00		36	ρ + τ	−0.56	0.31	17.12	0.00
	27	ρ	0.63	0.39	24.65	0.00		59	τ	−0.13	0.02	0.68	0.41
NDVI	22	τ	0.66	0.43	28.78	0.00	TVI	44	ρ + τ	0.48	0.23	11.46	0.00
	41	ρ + τ	0.51	0.26	13.30	0.00		51	τ	0.35	0.12	5.35	0.03
	58	ρ	0.16	0.03	1.00	0.32		61	ρ	0.13	0.02	0.67	0.42
PSNDa	25	τ	0.64	0.41	26.88	0.00	VOG	2	***τ***	0.80	0.64	68.85	0.00
	46	ρ + τ	0.47	0.22	10.87	0.00		8	***ρ + τ***	0.76	0.58	52.70	0.00
	62	ρ	0.12	0.01	0.56	0.46		21	ρ	0.68	0.46	32.31	0.00

Determination (R^2^) and correlation (COR) coefficients, as well as significance level of the relation between the 22 vegetation indices (abbreviations in Table 1) and destructively-measured chlorophyll content. Vegetation indices were calculated based on reflectance (ρ), transmittance (τ) or combination of ρ with τ (i.e., ρ + +τ) source images. Indices were ranked based on the R^2^ value (all three source images were included in the ranking). The entire leaf surface was averaged (n = 40). All four species (maize, rapeseed, barley and tomato) were plotted together. Plants were grown under control or deficient nitrogen levels. Bold text indicates R^2^ values greater or equal to 0.5.

Out of 66 indices, a significant and positive correlation (*P* < 0.001; R^2^ ≥ 0.5) between calculated chlorophyll content and destructively measured content was found in 25 and 18 indices for logarithmic and linear regression, respectively (bold highlighted in Tables 2 and 3). Importantly, 23 (out of the 25) and 17 (out of the 18) indices for logarithmic and linear regressions, respectively, were computed from τ and ρ + τ source images (bold italic highlighted in Tables 2 and 3). These results indicate that the additional τ measurements *per se* or in combination with ρ (i.e., ρ + τ) result in higher correlation coefficients between measured and estimated chlorophyll content.

Logarithmic regressions always gave higher correlation coefficients, as compared to linear regression models (Tables 2 and 3). Previous work also reports that indices are non-linearly related to chlorophyll content [31]. The highest correlation coefficients (i.e., 0.63 ≤ R^2^ ≤ 0.72) were noted in the logarithmic regression models of the following five indices: ρ + τEVI, τmND, ρ + τmND, τVOG, and ρBGI2 (Table 2, and Additional file 1: Figure S1). Importantly, the good correlation of these five indices with the destructively-measured values was driven by differences in chlorophyll concentration, owing to both the growth nitrogen regime and species.

Indices developed for carotenoids’ pigment changes (i.e., SIPI, PSRI) showed a poor relationship with chlorophyll content (i.e., R^2^ ≤ 0.52; Tables 2 and 3). Indices, traditionally used for vegetation monitoring (i.e., NDVI, SR, mSR), did not obtain as good results as red edge and combined indices (Tables 2 and 3). For NDVI specifically, its low performance might be explained by the fact that it changes only when chlorophyll concentration do not exceeds the value of 10 μg cm^−2^ [31,46]. In this study, chlorophyll concentration was eight- to nine-fold higher than this value. Indices developed for maximal sensitivity to leaf area index (i.e., MACRI, TCARI, TCARI/OSAVI) presented low correlation coefficients (i.e., R^2^ ≤ 0.44; Tables 2 and 3). This is because the aforementioned indices are not sensitive to changes in chlorophyll content at low leaf area index values [43], as those of this study where a set of a few leaves were assessed.

Interestingly, indices based on the off-chlorophyll absorption centre wavebands (690–730 nm; e.g., mND, VOG) seemed to perform better than indices based on the main chlorophyll and carotenoids’ absorption regions (450–550 and 670–680 nm, respectively; e.g., NDVI, MCARI) [47,48].

Besides the optical indices, employed here, there are also other approaches to estimate chlorophyll content by using spectral signature [49-51]. In this paper, we look into the first methodology, due to the simplicity of computation and implementation in studying plant properties. However, the performance of the HyperART, when using the aforementioned more elaborate approach, stays unexhausted and necessitates further work.

## Conclusion

The HyperART system was designed to provide data with high spectral and spatial resolution, which can be readily employed for phenotypic measurements, such as disease detection and quantification of chlorophyll content. The newly-introduced HyperART system scans entire leaves, attached to the plants, whereas currently-used instruments (e.g., FluoWat) are limited to spot (point) measurements. The system measures both reflectance (ρ) and transmittance (τ) data, by which the absorption (α) spectrum can be calculated, with one sensor. In this study we demostrated that HyperART measurements were consistent with data acquired with the well-established FluoWat device. We were able to show that the estimation of chlorophyll content of entire leaves, sampled from four different crops, was reasonably accurate. Moreover, it was shown that the system leads to an improved detection and quantification of disease symptoms (model plant-pathogen interaction *Cercospora beticola* and *Beta vulgaris* spp. *vulgaris*). Finally, it was noted that the non-absorbed radiation signal (i.e., ρ + τ) reduced data noise in disease detection and significantly improved the estimation of leaf chlorophyll content across species with different leaf structure. Based on our results, we suggest that the HyperART system holds a high potential for studying plant responses to both biotic and abiotic stressors at the entire leaf level.

## Methods

### Plant material and growth conditions

Two experiments were conducted, each case study focused on another set of species. Both experiments took place in a greenhouse located in the western part of Germany (Jülich, 50°N; 6°E).

#### Experiment I: Cercospora beticola infection

Two single plants of a sugar beet line (*Beta vulgaris* ssp. *vulgaris*; identification number 8RF5006 (HS) KWS SAAT AG, Einbeck, Germany) were cultivated. During growth, air temperature and relative air humidity were 20.9 ± 6.9°C (range: 8.5–47.6) and 59.6 ± 21.7% (range: 8.2–96.9), respectively. Supplementary light was provided by high-pressure sodium lamps (SON-T Agro, 250 W, Philips, Eindhoven, The Netherlands), at 200 μmol m^−2^ s^−1^ photosynthetic photon flux density for 16 h per day. Plants were grown in 1 L pots, filled with soil mixture (1:1, v/v) of Hawita Dachstaudensubstrat (HAWITA GRUPPE GmbH, Vechta, Germany) and Pikiererde (Einheitserde Typ P Pikier, Balster Einheitserdewerk GmbH, Frödenberg, Germany). Plants were kept well-watered, and were weekly provided with nutrient solution (0.2%; Hakaphos Blau, COMPO GmbH & Co. KG, Münster, Germany) starting from the second week following emergence. The amount of nutrient solution was 60 mL per plant (up to 4 weeks following emergence), and was subsequently increased to 80 mL. Plants were grown for six weeks (starting from 17^th^ of July, 2012) up to the developmental stage 18 of the BBCH scale [52].

Before inoculation, the pathogen *Cercospora beticola* was grown on agar plates (1.5%; Sigma-Aldrich Chemie GmbH, Taufkirchen, Germany), containing 50% vegetable juice (Gemüsemix, Eckes-Granini Deutschland GmbH, Nieder-Olm, Germany). Pathogen cultivation took place in a growth chamber, set at 60% relative air humidity and 26°C air temperature. The pathogen was kept on the agar plates for 3 weeks. To stimulate conidia production, plates were placed under ultraviolet light (340–400 nm) for 3 days before conidia collection. Subsequently, conidia were scraped with a slide, and were suspended to an aqueous solution containing Tween20 (0.1%; Sigma-Aldrich Chemie GmbH, Taufkirchen, Germany). The solution concentration was set to 3 × 10^4^ conidia mL^−1^ using a hemocytometer (Thoma chamber, Carl Roth GmbH + Co. KG, Karlsruhe, Germany). Plant inoculation was conducted, as previously described by Schmidt *et al.* (2008) [53]. Following inoculation, plants were kept at 80–100% relative air humidity for one week. During that time, air temperature was 24 ± 0.3°C (range: 15–35). Light intensity was set to 300 μmol m^−2^ s^−1^ one day after inoculation. The control plants were inoculated with an aqueous solution containing only Tween20 (0.1%). Measurements were conducted about three weeks after inoculation, when symptoms of *Cercospora beticola* infection were visible.

#### Experiment II: Nitrogen deficit

Two dicotyledonous crops [rapeseed (*Brassica rapa* cv. Campino) and tomato (*Lycopersicon esculentum* cv. Harzfeuer)] and two monocotyledonous crops [maize (*Zea mays* cv. Gelber) and barley (*Hordeum vulgare* cv. Barke)] were grown under controlled conditions in a greenhouse. During growth period, air temperature and relative air humidity were 21.1 ± 0.3°C (range: 19.7–23.9) and 51 ± 1% (range: 43–60), respectively. Supplementary light was provided by high-pressure sodium lamps, as described above, at 50 μmol m^−2^ s^−1^ photosynthetic photon flux density for 16 h per day (from 0600 to 2200 hours). The average daily light integral, including natural light, was 9.6 ± 0.5 mol m^−2^ day^−1^. Seeds were sown and germinated in small pots until plants were grown up to the cotyledons’ stage (i.e. fully open cotyledons and before the appearance of the first leaf for dicotyledonous species and the second leaf stage for monocotyledonous species). Subsequently, seedlings were transplanted into 17.4 L rectangular boxes containing a ready-made soil mixture (‘Nullerde’ Archut Erzeugnisse GmbH, Vechta, Germany) with low nitrogen content (50 mg L^−1^). A planting density of five plants per box was used. Boxes were randomly distributed on a glasshouse table.

After planting, plants were exposed to different levels of nitrogen fertilisation. For this, eight boxes, containing five plants each, were separated into two different treatments: control (10 mM nitrogen concentration) and nitrogen deficit (15% of the control). Once a week, each box received 500 mL of nutrient solution [54]. The experiment lasted for 23 days, following planting (starting from 25th of April, 2013).

### Point measurements

The spectral signature of selected leaves was also obtained by using a high spectral resolution point spectroradiometer (ASD FieldSpec® 3, Analytical Spectral Devices, Boulder, CO, USA), operating at a spectral range between 350 and 2500 nm. The nominal spectral resolution is 3 and 10 nm FWHM in the 350–1050 and 1050–2500 nm regions, respectively. The sampling interval is 1.4 and 2 nm in the 350–1050 and 1050–2500 nm regions, respectively. The FieldSpec fibre optic was connected to a FluoWat leaf clip [17,18]. The FluoWat leaf clip offers the possibility to measure both reflectance and transmittance of either sample side. Illumination was provided by a cold light source (Schott KL 1500 LCD 150 W, Lighting and Imaging SCHOTT AG, Mainz, Germany). Due to the spectral output of the illumination source, spectral readings were limited to the 400 to 1700 nm range. The intensity of the electromagnetic radiation emission was determined by using a 99.9% reflective white standard (Spectralon, Labsphere Inc., North Dutton, NH, USA). The spectra of the samples were divided by the white standard spectra and multiplied by a correction factor (provided by Labsphere Inc.) for calculation of the relative values. In all measurements, leaf reflectance was assessed on the adaxial (upper) surface.

### Spectra comparison

To assess the validity of the spectral data acquired using the HyperART system (first prototype), simultaneous measurements using both this and the FluoWat device were performed on three disease-free sugar beet leaves from the control plant. Five FluoWat point measurements were averaged, and these were tested against five comparable points that were selected in the hypespectral image obtained by using the HyperART system. In this study we combine the spectra of two cameras by selecting nearly the same regions in both hyperspectral images and averaging them. Since the investigated plant tissue was homogeneous, and thus signal changes spatially smooth, we assume that the spectral differences do not change much when the regions of interest do not fit perfectly.

The sensors of these two compared instruments have similar spectral resolution, whereas spectral sampling differs. For comparison of the spectra obtained by the two instruments, the data were converted to a similar spectral sampling. This was done by reducing the FieldSpec spectral sampling to the one of the line scanners, by matching the wavelength.

### Quantification of *Cercospora beticola* symptoms

The potential of the HyperART system in detecting and quantifying the symptoms of *Cercospora beticola* infection, on sugar beet leaves was evaluated. Three leaves of an infected plant and three leaves of an infection-free plant, were placed in the leaf clip between the mirrors of the HyperART system (first prototype). Two leaves of the infected plant had necrotic areas, whereas the third one did not have any disease symptoms. Disease severity was also estimated visually (expressed in percentage) for each leaf.

The background of the hyperspectral image was masked out using a threshold in the EVI image. After image processing, a disease specific index $\Big( Cercospora\kern0.5em  leaf\  spot\  index\ (CLSI)=\frac{\uprho 698-\uprho 750}{\rho 698+\rho 750}-\rho 734$; [13]) was calculated from the ρ, τ and ρ + τ data, respectively. Low values indicate low disease severity, and vice-versa. From the CLSI images, histograms with a binning of 0.01 were computed and divided by overall number of pixels. By this the normalized histograms represent estimated probability density function of CLSI values. As an indicator of how well the two modes can be distinguished, we calculated the so-called bimodal amplitude (referred as A_b_). The bimodal amplitude equation (${A}_b=\frac{A_m-{A}_v}{A_m}$; [55]) was used to analyse the CLSI histograms. For calculating A_b_, the smaller peak amplitude (referred as A_m_) and the amplitude of the minimum between the two peaks (referred as A_v_) were computed. Peak heights (normalized frequencies) and locations were 0.021 at ρCLSI −1.21 value, 0.022 at τCLSI −1.32 value and 0.014 at ρ + τCLSI −1.42 value. The minima normalized frequencies were 0.005 at −0.75 value, 0.004 at −0.75 value, and 0.004 at −0.75 value for ρCLSI, τCLSI and ρ + τCLSI, respectively. A_b_ values close to one indicate a better double peak distinction.

In order to evaluate the separability performance in the index images of ρCLSI, τCLSI and ρ + τCLSI, these were firstly classified by using the K-Means unsupervised classification (ENVI 4.8; [56]). The automatic algorithm was set to separate the data in the following two classes: infected and non-infected leaf tissue. The same classes were used for a supervised classification performed by support vector machine on the ρ, τ and ρ + τ hyperspectral images. Therefore, symptomatic and symptom-free regions were selected manually in the hyperspectral images. After all images were classified, the validity of unsupervised classification performance was evaluated using post classification. Unsupervised classification results for the index images of ρCLSI, τCLSI and ρ + τCLSI were compared by confusion matrix with the respective supervised classification (ENVI 4.8). The supervised classification results were used as ground true images.

### Vegetation indices

Twenty two commonly-used indices for chlorophyll content estimation at both leaf and canopy levels (Table 1) were applied to ρ, τ, and ρ + τ pre-processed hyperspectral images. Throughout the paper, the letters ρ, τ, and ρ + τ before an index indicate the source of the hyperspectral image, which was used for the calculation. Sixty six outcomes (i.e., 22 indices × 3 sources) were obtained. Both a simple linear regression and a logarithmic model were applied to the relationships between predicted (by the indices) and destructively-measured (described below) chlorophyll content.

### Assessment of chlorophyll content

Sampling for biochemical assessment of chlorophyll content was conducted 23 days after planting. The third and fourth (counting from the apex) fully-expanded leaves were collected for rapeseed, barley and tomato (two leaves per plant; n = 10). The middle portion of the third fully-expanded was sampled for maize (one leaf per plant; n = 5). Chlorophyll concentration was assessed in the same leaves, where hyperspectral measurements were done. Non-destructive (hyperspectral imaging) and destructive (chlorophyll content) measurements were performed within 24 h.

Collected plant material was immediately frozen in liquid nitrogen, and stored at −80°C for further analysis. Plant material was homogenized by grinding the tissue in liquid nitrogen. About 50 mg of the homogenized sample were ground in 6 ml acetone (100% pure solvent), by using a mortar and pestle. Since chlorophyll is light sensitive, extraction took place in a dark room. Liquid nitrogen was continuously used to cool down the sample. The resulting homogenate was centrifuged in test tubes for 6 min for precipitation of the cell debris. Absorption spectra of the supernatants were recorded. Chlorophyll content was calculated by equations described by Lichtenthaler [57].

## Additional file

Additional file 1: Figure S1. Logarithmic relationships between calculated indices and destructively-measured chlorophyll content. The leaves were sampled from four different crop species. The green colour indicates control plants, while the red colour refers to the nitrogen-deficient plants. Indices were calculated based on reflectance (ρ), transmittance (τ) or combination of ρ with τ (i.e., ρ + +τ). Spectra were acquired by using the hyperspectral absorption reflectance transmittance imaging (HyperART) system. Abbreviations are explained in Table 1.

## Abbreviations

α: Absorption
CLSI: *Cercospora* leaf spot index
A_b_: Bimodal amplitude
HyperART: Hyperspectral absorption-reflectance-transmittance imaging
NIR: Near infrared
PAR: Photosynthetic active radiation
ρ: Reflectance
τ: Transmittance
