# Supplementary figures and images for: HyperART: non-invasive quantification of leaf traits using hyperspectral absorption-reflectance-transmittance imaging
Source: Plant Methods. 2015 Jan 16;11:1. doi: 10.1186/s13007-015-0043-0 (PMC4302522; doi:10.1186/s13007-015-0043-0)

Index calculated from:

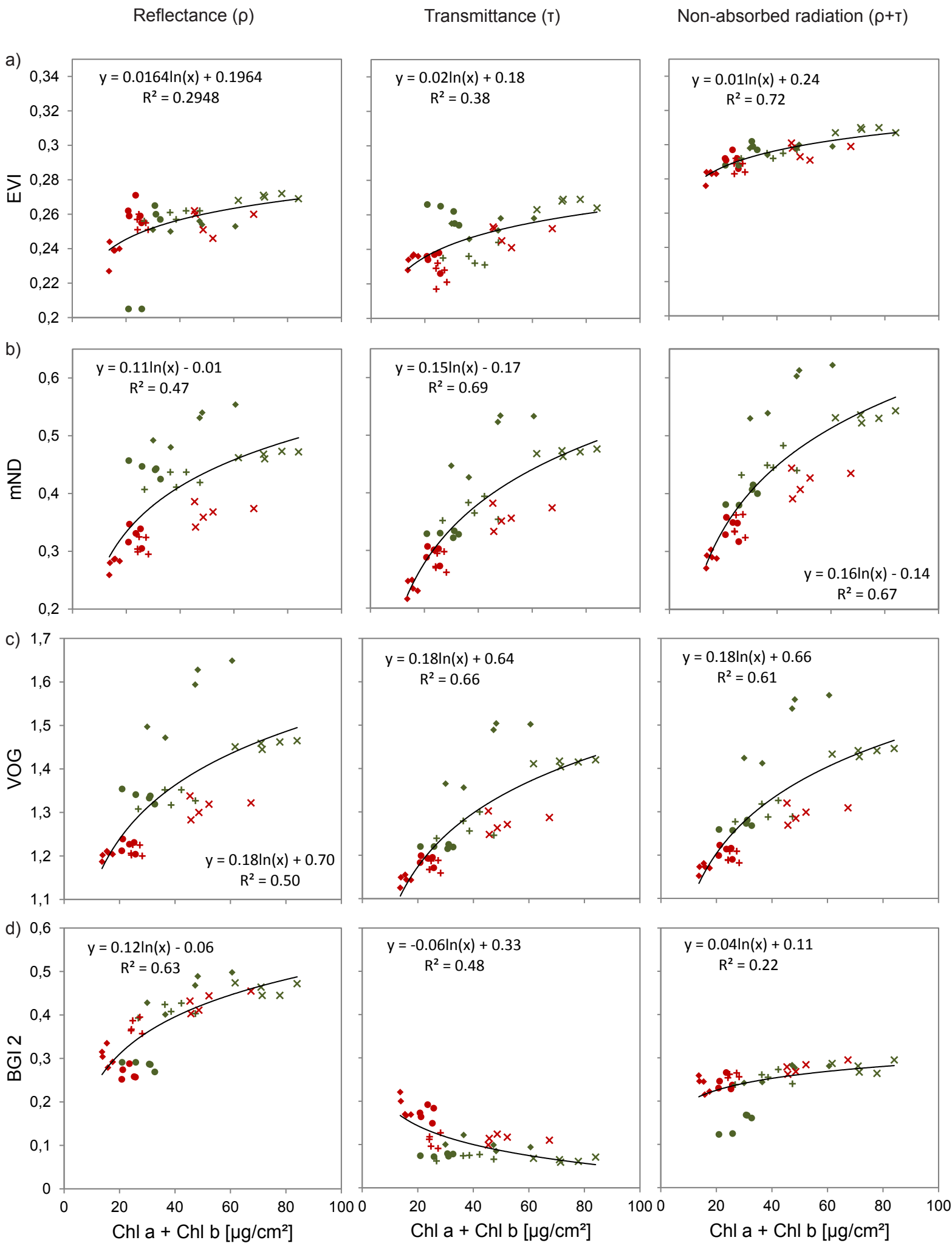

◆ Maize + Tomato ● Canola × Barley

Supplement: Additional file 1: Figure S1. — Logarithmic relationships between calculated indices and destructively-measured chlorophyll content. The leaves were sampled from four different crop species. The green colour indicates control plants, while the red colour refers to the nitrogen-deficient plants. Indices were calculated based on reflectance (ρ), transmittance (τ) or combination of ρ with τ (i.e., ρ + +τ). Spectra were acquired by using the hyperspectral absorption reflectance transmittance imaging (HyperART) system. Abbreviations are explained in Table 1. [file 13007_2015_43_MOESM1_ESM.pdf]
